# Assessment of characteristics of patients with cholelithiasis from economically deprived rural Karachi, Pakistan

**DOI:** 10.1186/1756-0500-5-334

**Published:** 2012-06-28

**Authors:** Muhammad Naeem, Nasir Ali Rahimnajjad, Muhammad Kazim Rahimnajjad, Madiha Khurshid, Qazi Jalaluddin Ahmed, Syed Mariam Shahid, Faiza Khawar, Molham Mustafa Najjar

**Affiliations:** 1 Dow Medical College, Dow University of Health Sciences, Karachi, Pakistan; 2 Chief Resident, Department of Orthopaedic Surgery, Liaquat National Hospital, Karachi, Pakistan; 3 Associate professor and Chairperson, Department of General surgery, KVSS Hospital, Karachi, Pakistan

**Keywords:** Gall stones, Risk factors, Pakistan

## Abstract

**Background:**

Gallstones have been regarded as one of the most expensive diseases in Gastroenterology, posing a great economic burden on developing nations. The majority of Pakistani people live in rural areas where healthcare facilities are not available or are very primitive. We aim to assess the characteristics among cholelithiasis patients from rural Karachi so that a prevention campaign can be launched in rural underprivileged settings to reduce the economic burden of this preventable disease.

**Method:**

A total of 410 patients were included in the study after giving verbal consent as well as written consent. Variables such as age, weight, height, body mass index (BMI), blood pressure, waist circumference**,** number of children, monthly family income, number of siblings, and number of family members, were considered in this questionnaire. All data was analysed by SPSS ver. 16.0. Mean and standard deviation (SD) were calculated for continuous variables. Frequency and percentages were calculated for categorical variables.

**Results:**

Nearly 85.4% of the participants were female. The mean ± S.D. for age was 43.8 ± 9.59. Nearly 61% of the patients were illiterate. All of our patients were from low socioeconomic status and their mean salary ± S.D. was 6915 ± 1992 PKR (1 US $ = 90.37PKR). 75% of them were smokers with mean consumption ± S.D. of 7.5 ± 10 cigarettes per day. Fibre in diet was not used by 83.65% of patients. 40.2% were living in combined families. 61% were living in purchased homes. A positive history of diabetes mellitus was given by 45.1%, family history of cholelithiasis by 61% and history of hypertension by 31.7% of subjects. Soft drink consumption was given by 45.1% of patients; while only 8.5% used snacked daily. Tea was consumed by 95.1% of the subjects. Daily physical activity for 30 minutes was reported by only 13.4% of participants.

**Conclusion:**

In conclusion, rural dwellers from low socioeconomic strata are neglected patients and illiteracy further adds fuel to the fire by decreasing the contact with the health professionals. Assessment of the characteristics are very important because considering the great socio-economic burden, an intervention strategy in the form of mass media campaign as well as small group discussions in such rural areas can be formulated and applied to high risk populations to reduce the burden and complications of gallstone disease.

## Background

Gallstones are the concretions that can form in any part of the biliary tract, and when this involves the gall bladder, it is called cholelithiasis. Gallstones are one of the most prevalent and most expensive gastroenterological diseases, leading to a great economic burden. Annually 600,000 cholecystectomies are performed in 10-15% of the American adult population, and an estimated $5 billion is being spent annually on the treatment of gallstones, while the complications of the surgery consume nearly $6.5 billion US. [1-3] Data from Pakistan is still insufficient, but previous study has found the surgical incidence of 9.03% from southern Sindh area of Pakistan [4].

A well-known mnemonic for memorizing the risk factors associated with gallstones is female, fat, fertile and forty; which has been proven by various studies. Previously described risk factors include age [5-7], female gender [8,9], obesity [1,10], high cholesterol intake [11], decreased fibre intake [12], smoking [13], high parity [14], a family history of gallstones and decreased physical activity [9]. Gallstone disease is regarded as a surgical disease since only a cholecystectomy is the cure, but by identifying possible risk factors this could help in designing therapeutic as well as preventive strategies [15]. Female gender and advancing age are non-modifiable risk factors for gallstones. However, keeping in mind the great socio-economic burden of this disease, and that the majority of Pakistani people live in rural areas where healthcare facilities are not available or are very primitive. We aim to assess the characteristics among cholelithiasis patients from rural Karachi so that a prevention campaign can be launched in rural underprivileged settings to reduce the economic burden of this preventable disease.

## Methods

### Participants and setting

This was a cross sectional study conducted at the two largest tertiary care government-ran teaching hospitals of Karachi, Civil hospital and Jinnah Postgraduate Medical Centre, from November 2008 until June 2010. These two hospitals provide tertiary care facilities free of cost, so they are the primary care providers for the rural dwellers of the city who cannot afford the expensive treatments at other hospitals. 410 participants who were the rural dwellers from the Karachi city area were enrolled in the above-mentioned period through the outpatient department (OPD) of the respective hospitals, and were diagnosed on history, physical examination, and ultrasound examination. All patients were having symptomatic gallstones and all of them underwent ultrasound examination. No patient was excluded. Each patient gave the verbal consent for participation; while written consent was taken prior to surgical procedure as part of the informed consent, and the name of the patient and other identifying information was not included in the questionnaire; therefore, full confidentiality was ensured. The Departmental Ethical Review Board of KVSS Hospital gave ethical approval. Information was gathered, through detailed questionnaires, which were completed by trainee postgraduate doctors in the OPD, as well as on the day of surgery to ensure the validity of the data. In order to check the recall bias in regards to diet, we kept our subjects for forty-eight hours under observation and cross checked the data with our observations.

### Dietary habits

**Figure 1:**
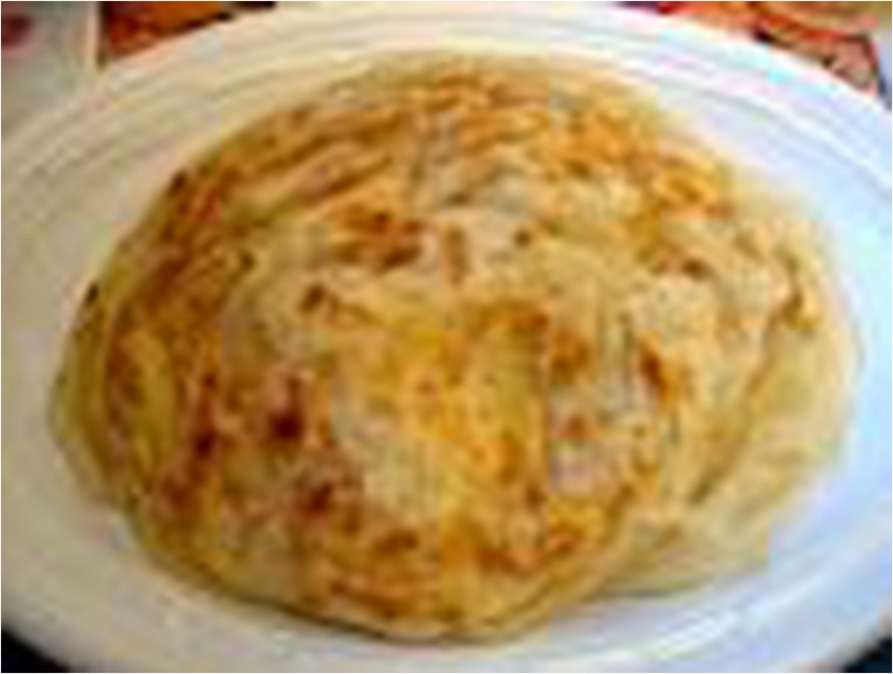
Paratha (A flat bread made of wheat flour fried in pan in ghee).

**Figure 2:**
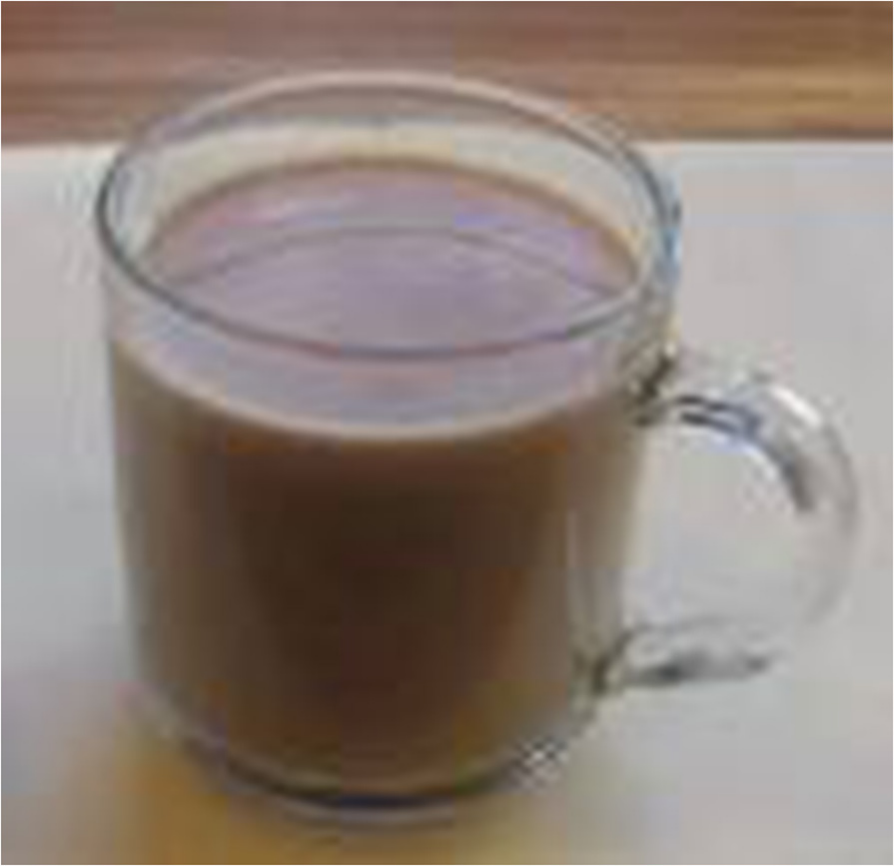
Tea (locally called chai and is consumed more than two times/day).

**Figure 3:**
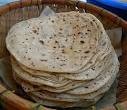
Roti (A flat bread made of wheat flour but is not fried).

**Figure 4:**
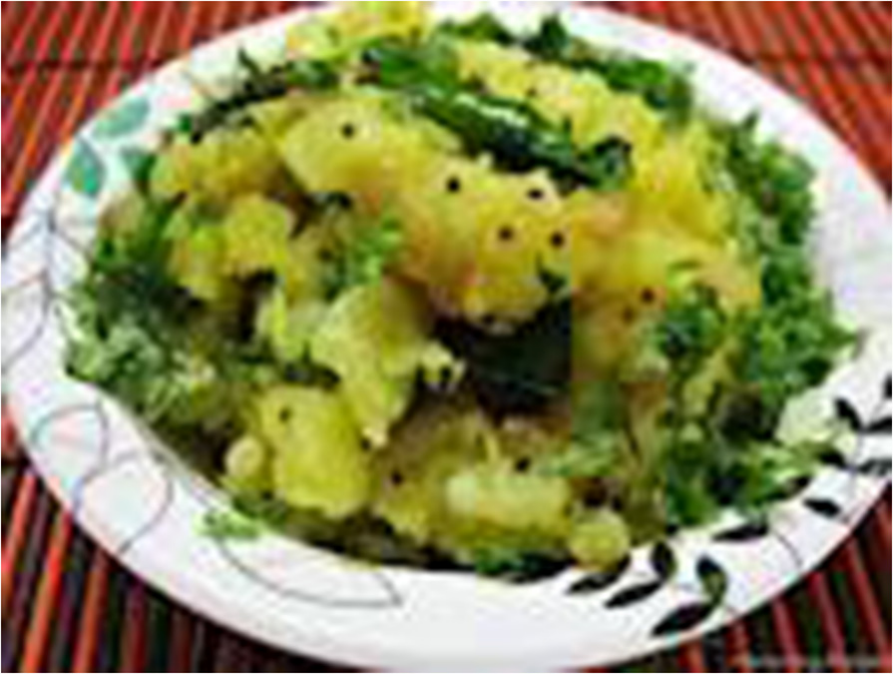
Aaloo Curry (Curry made by potato).

Pakistan is a developing country with most of its population residing in rural areas and majority are living in poor conditions. Diet among our participants is consumed as breakfast and dinner; due to rising economic crises most of people skip lunch. The diet consumed is high in carbohydrate contents. Refined flour is used primarily which lack fibers. Breakfast typically consists of Tea (locally called CHAI) and Paratha (A flat bread made of wheat flour fried in pan in ghee). People mostly cannot afford the meat therefore dinner consists of roti (A flat bread made without ghee) from refined flour or refined rice with curry mostly made by potato along with locally available vegetables. The food is cooked on high temperature for prolong duration which destroys the essential ingredients of the food. These dietary habits on one hand reflect the poor socio economic status of the people and on other hand reflect the increase predisposition to serious diseases. These foods are shown in Figure 1, 2, 3 and 4.

### Measurements

After the completion of the questionnaire, weight and height measurements were obtained for each participant. The patients’ were weighed fully clothed with the exception of shoes using a digital scale, and the weight was then rounded to the nearest 0.05 kg. Height was measured to the nearest 0.5 cm using a height meter after he patient removed their shoes, and placed his or her heels together. Blood pressure was taken using a mercury sphygmomanometer (Bokang. CE 0483) which measures blood pressure to the nearest 10 mm. Hg. BMI was calculated using Asian cut-off values.

### Variables in study

There were two sets of data. One was continuous, and the other was non-continuous data. The continuous data variables were age, weight, height, BMI, blood pressure, waist circumference**,** number of children, monthly family income, number of siblings, and number of family members. The non-continuous variables were sex, marital status, qualification, occupation, if the home was owned or rented, ethnicity, addiction and substance, family history of Cholelithiasis, history of diabetes, history of hypertension, cooking preparation, soft drink use, snack consumption, tea use, and physical activity.

### Statistical analysis

We used Statistical Software for Social Sciences (SPSS version 16.0) for statistical analysis. Descriptive analyses were performed to investigate the distribution of our data. Mean and standard deviation (SD) were calculated for continuous variables. Frequency and percentages were calculated for categorical variables. Weight of the individuals was categorized into underweight, normal weight, overweight, and obese by using the South Asian cut-off for BMI. Individuals were labelled as underweight if BMI was less than 18.5, normal if BMI was between 18.5 to 23.99, overweight if BMI was between 24 to 26.99, and obese if BMI is greater than or equal to 27. Individuals were labelled as hypertensive if his or her diastolic blood pressure was found to be greater than or equal to 90 mmHg, or if his or her systolic blood pressure was found to be greater than or equal to140 mmHg.

## Results

**Figure 5:**
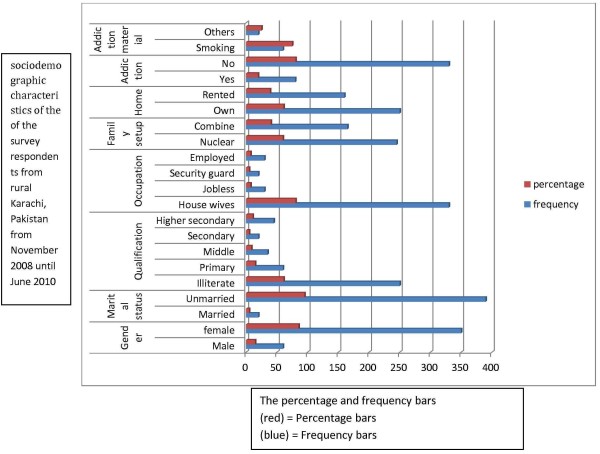
Bar diagram showing the sociodemographic characteristics of the of the survey respondents from rural Karachi, Pakistan from November 2008 until June 2010.

**Figure 6:**
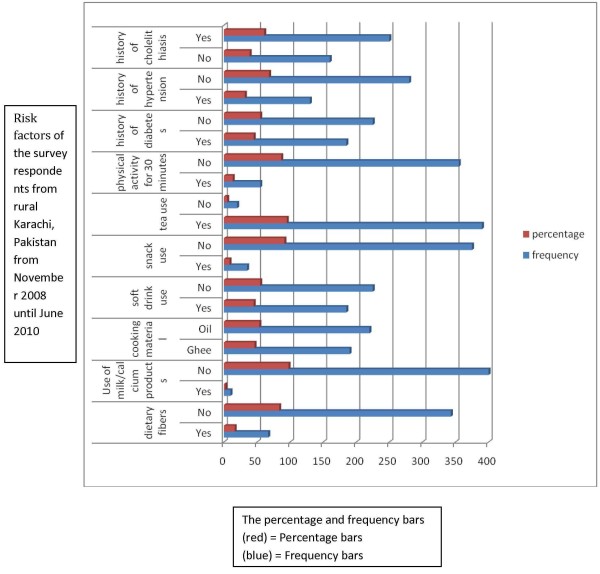
Bar diagram showing the frequency of Risk factors of the survey respondents from rural Karachi, Pakistan from November 2008 until June 2010.

The socio-demographic determinants are given in Table 1 and also in Figure 5 along with risk factors frequencies for cholelithiasis in Table 2 and also in Figure 6. Table 3 shows the measured characteristics of the participants and Table 4 shows blood pressure and BMI profiles. Nearly 85.4% of the participants were female. The mean ± S.D. for age was 43.8 ± 9.59 (95%C.I. 42.87 — 44.72). 95.1% of the subjects were married. Nearly 61% of the patients were illiterate, and the remaining included primary (14.6%), middle (8.5%), secondary (4.9%) and post-secondary (10.9%) education. Since 85.4% of the participants were female, the main occupations were housewives (80.5%), jobless (7.3%), security guards (4.9%) and private and government employees (7.3%). All of the patients were from low socio-economic status; therefore, the mean salary ± S.D. was 6915 ± 1992 PKR and 95%C.I. was 6722.20―7107.80 (1 US $ = 90.37PKR). Only 19.5% (100% of males and 4.9% of females) of the patients were addicted to tobacco in various forms and alcohol and of those who were addicted, 75% of them were smokers with mean consumption ± S.D. of 7.5 ± 10 cigarettes per day. Dietary fibres were not used by 83.65% of patients based on their recall of past seventy-two hours, as well as our forty-eight hours observation. In regards to family setup, 40.2% were living with their extended family. 61% were living in their own houses, and 39% were living in rented units. Nearly all the patients were from low socio-economic slum areas where an individual does not have a legal title to the land. Calcium in the form of milk or supplements was used by only 2.31% of the respondents. Positive history of diabetes mellitus was reported by 45.1%. A family history of cholelithiasis was seen in 61% of patients, while a history of hypertension was reported 31.7% of subjects. The patients stated the cooking material most often used was ghee (46.3%), and vegetable oil (53.7%). Soft drink consumption was reported by 45.1% of patients; while only 8.5% consumed snacks daily. Tea was drunk by 95.1% of the subjects. 30 minutes of daily physical activity was reported by only 13.4% participants.31.7% of participants were hypertensive (cut off value 140/90 mm. hg.). Asian cut off values for BMI showed no participant was underweight, 13.4% normal, 22% overweight and 64.4% clinically obese. 95% C.I. for BMI was 29.49―31.11. Mean parity ± S.D. was 5.42 ± 2.

**Table 1:** Sociodemographic characteristics of the survey respondents from rural Karachi, Pakistan from November 2008 until June 2010 (n = 410)

**Variable**		**Frequency**	**Percentages**
**Gender**	Male	60	14.6
	Female	350	85.4
**Marital Status**	Unmarried	20	4.9
	Married	390	95.1
**Qualification**	Illiterate	250	61.0
	Primary	60	14.6
	Middle	35	8.5
	Secondary	20	4.9
	Higher/post secondary	45	10.9
**Occupation**	House wives	330	80.5
	Jobless	30	7.3
	Security guard	20	4.9
	Employed	30	7.3
**Family setup**	Nuclear	245	59.8
	Combine	165	40.2
**Home**	Own	250	61.0
	Rented	160	39.0
**Addiction**	Yes	80	19.5
	No	330	80.5
**Addiction Material (out of 80)**	Smoking	60	75.0
	Others	20	25.0

**Table 2:** Risk factors of the survey respondents from rural Karachi, Pakistan from November 2008 until June 2010 (n = 410)

**Risk Factors**		**Frequency**	**Percentages**
**Dietary fibers**	Yes	67	16.34
		No	343	83.65
**Use of Milk/Calcium Products**	Yes	10	2.31	
	No	400	97.69	
**Cooking Material**	Ghee	190	46.3	
	Oil	220	53.7	
**Soft Drink Use**	Yes	185	45.1	
	No	225	54.9	
**Snack Use**	Yes	35	8.5	
	No	375	91.5	
**Tea Use**	Yes	390	95.1	
	No	20	4.9	
**Physical activity for 30 minutes**	Yes	55	13.4	
	No	355	86.6	
**History of Diabetes**	Yes	185	45.1	
	No	225	54.9	
**History of Hypertension**	Yes	130	31.7	
	No	280	68.3	
**History of Cholelithiasis**	No	160	39.0	
	Yes	250	61.0	

**Table 3:** Measurement of characteristics of of the survey respondents from rural Karachi, Pakistan from November 2008 until June 2010 (n = 410)

**Variables**	**Mean ± S.D.**	**95% C.I.**
**Weight (Kg)**	59.56 ± 8.71	58.71 — 60.40
**Height (inches)**	56.98 ± 6.02	56.39 — 57.56
**BMI**	30.30 ± 8.38	29.49 — 31.11
**Waist circumference (inches)**	34.14 ± 3.25	33.82 — 34.46
**Systolic Blood Pressure (mmHg)**	126.21 ± 14.49	123.46 — 128.96
**Diastolic Blood Pressure (mmHg)**	80.46 ± 7.59	79.73 — 81.19
**Age**	43.8 ± 9.59	42.87 — 44.72
**No. of children**	5.42 ± 2.0	5.23 — 5.61
**Monthly income (In Rupees)**	6915 ± 1992	6722.20 — 7107.80
**No. of siblings**	6.88 ± 3.30	6.56 — 7.19
**No. of cigarettes**	7.50 ± 10.0	6.53 — 8.46
**Total family members**	7.08 ± 2.04	6.39 — 7.76

**Table 4:** Hypertension and BMI profiles of the survey respondents from rural Karachi, Pakistan from November 2008 until June 2010 (n = 410)

**Parameter**		**Frequency**	**Percentage**
**Hypertension**	Normotensive	280	68.3
	Hypertensive	130	31.7
**BMI**	Underweight	0	0
	Normal	55	13.4
	Overweight	90	22
	Obese	265	64.4

## Discussion

Global prevalence of this disease is very high in developed countries, but developing countries such as Pakistan are currently facing the rapidly increasing burden of gallstone disease as well, due to the over-consumption of fast food prevalent in these countries. This study was done in order to see what factors were found in relation to cholelithiasis in rural dwellers. In the United States, 20,000,000 cases of gallstones are reported annually, and in the United Kingdom, the incidences of gallstones are 8% and 20% for persons above 40 and 60 years respectively. Despite of this increase in Pakistan, little preventative work has been done to decrease the number of gallstone cases [16,17].

The occurrence of gallstones disease is positively related to advancing age, as gallstones are unusual in persons younger than 30 years [7]. In our study, mean age was 43.8 ± 9.59 years, age factor has been previously highlighted in several studies. As in Taiwan, In older persons, two indicators of gallstones were being over sixty years of age and being positive for diabetes mellitus [5]. Similarly, autopsy studies conducted in Sweden and the Czech Republic showed the incidence of gallstones to be 30% in men and 50% in women older than 20 years of age [6]. Our study also justifies The Wheeler Study, which showed significant association between marital status and gallstone occurrence, as 95.1% of our subjects were married [18]. Another significant parameter was that 85.4% of our patients were female, and it has been previously documented in many studies that being female is the single most important non-modifiable cause of gallstones [8,9]. The factor concerning the family history of cholelithiasis significantly deviated from what was presented by an international study. The international study stated that 39% of the patients whose first degree relative had suffered from cholelithiasis had gallstones. In comparison, our study suggests 61% of patients in our study have had a positive family history of cholelithiasis. This difference may have occurred because our study was only focusing on those patients who were presenting to the hospital with symptomatic gallstones; while that study covered a large number of people who were otherwise healthy [19].

How dietary factors influence the formation of gallstones is still unclear, but many studies have proven that dietary risk factors such as increased cholesterol intake, increased consumption of refined sugars, increased saturated fat intake, tamarind, consumption of high glycemic index foods, decreased calcium intake, and low dietary fibre intake are risk factors for gall stones. Many studies showed that fibre, especially bran, can reduce the incidence of gallstones. In our study, we found relation between low calcium intake [20], low dietary fibre [12], and increased saturated fat intake [11] and gallstone occurrence. Calcium has been postulated in altering the bile composition by preventing the reabsorption of secondary bile acids in the colon, whereas sugars, by altering lipoprotein metabolism, may influence the formation of gallstones. We excluded tamarind because it is a highly common ingredient in most Pakistani dishes. Coffee has shown to decrease gallstone disease by increasing the enterohepatic circulation of bile acids [9], but we have excluded this factor too because in Pakistan, coffee is not consumed among lower socio-economic groups. However, 95.1% of the subjects consumed tea three times daily. How the consumption of tea effects gallstone formation has not been concluded.

Regarding addictions, only 19.5% of the subjects were addicted to various form of tobacco and alcohol (100% males and only 4.9% females) and of those who were addicted, 75% smoked cigarettes. Since our study subjects were predominantly female, and in Pakistani culture females do not smoke, 100% of subjects who smoked were male. Our study supports a Denmark study [9], which associated smoking as risk factors for men and not for women. Some authors found significant association between number of cigarettes smoked and occurrence of gallstones, while others did not find any causal relationship. Smoking is associated with low plasma HDL, which itself increases the risk for gallstones. Smoking also hampers prostaglandins and mucus production in the gallbladder, which predisposes one to gallstones [13].

In a Danish study, it was concluded that increased BMI and slimming treatments were associated with gallstones [10]. In our study, 22% of the subjects were overweight, and 64.4% were clinically obese.

Unhealthy lifestyle and decreased physical activity were also major risk factors for gallstones. It has been proven that 34% of symptomatic gallstone disease in men could have been prevented by increasing endurance exercising to 30 minutes of training five times per week [21]. A study in Boston showed no significant association between gallstone disease and energy intake when adjusting for intake of cholesterol, animal fat, animal proteins, carbohydrates or sucrose [22]. Many studies have associated gallstones with a positive history of diabetes, but we did not find any significance, confirming the findings of Denmark study [10]. In Our study, we found high parity among the cholelithiasis patients as proved in a previous study and it has been proposed that during pregnancy, estrogen causes sluggish contractility, leading to the formation of gallstones [1].

## Conclusion

It is important to note that the major limitation of our study is the cross-sectional hospital based design, which is not meant to assess the risk factors. Rather, we have studied the frequencies of proposed risk factors, which were present in the survey respondents.

Despite common occurrence, not much work has been done in this regard from Pakistan, where majority of the population belongs to either lower socioeconomic status or rural area. Rural dwellers from low socioeconomic strata are neglected patients and illiteracy further adds fuel to the fire by decreasing the contact with the health professionals. Assessment of the characteristics during the first contact with the health professional are very important to nip the evil in bud, since these patients belong to areas that are dominated with traditional faith healers or noncertified doctors who have least interest in the prevention of diseases. Considering the great socio-economic burden, a prevention strategy to highlight the importance of prevention of these risk factors in the form of mass media campaign as well as small group discussions to stop smoking, to promote the consumption of dietary fibers and to promote physical activity in such rural areas can be formulated and applied to high-risk populations to reduce the burden and complications of gallstone disease.

## Competing interests

The authors declare that they have no competing interest.

## Authors’ contributions

MN, Conceived the study, Manuscript draft, study design. NAR, Manuscript Draft, statistical analysis. MKR, co-supervised the study and study design. MK, Statistical analysis and study design. QJA, Supervised the study and critical reviewing the manuscript. SMS, data collection, analysis and interpretation. FK, data collection, analysis and interpretation. MMN, data collection, analysis and interpretation. All authors read and approved the final manuscript.
